# Acute liver injury as a manifestation of granulomatous hepatitis: diagnostic challenges

**DOI:** 10.1093/omcr/omaf160

**Published:** 2025-09-15

**Authors:** Melissa N Martinez-Marquez, Sandra M Feria-Agudelo, Lumi G Alfonso-Zapata, German Barrientos-Cabrera, Jesús Ruiz-Manríquez, Froylan D Martínez-Sánchez

**Affiliations:** Department of Internal Medicine, Hospital General Dr. Manuel Gea Gonzalez, Calz. de Tlalpan 4800, Belisario Domínguez Secc 16, Tlalpan, 14080 Ciudad de Mexico, Mexico; Department of Internal Medicine, Hospital General Dr. Manuel Gea Gonzalez, Calz. de Tlalpan 4800, Belisario Domínguez Secc 16, Tlalpan, 14080 Ciudad de Mexico, Mexico; Department of Internal Medicine, Hospital General Dr. Manuel Gea Gonzalez, Calz. de Tlalpan 4800, Belisario Domínguez Secc 16, Tlalpan, 14080 Ciudad de Mexico, Mexico; Department of Internal Medicine, Hospital General Dr. Manuel Gea Gonzalez, Calz. de Tlalpan 4800, Belisario Domínguez Secc 16, Tlalpan, 14080 Ciudad de Mexico, Mexico; Department of Gastroenterology, Hospital General Dr. Manuel Gea Gonzalez, Calz. de Tlalpan 4800, Belisario Domínguez Secc 16, Tlalpan, 14080 Ciudad de Mexico, Mexico; Department of Internal Medicine, Hospital General Dr. Manuel Gea Gonzalez, Calz. de Tlalpan 4800, Belisario Domínguez Secc 16, Tlalpan, 14080 Ciudad de Mexico, Mexico; Facultad de Medicina, Universidad Nacional Autónoma de México, Escolar 411A, Copilco Universidad, Coyoacán, 04360 Ciudad de México, Mexico

**Keywords:** granulomatous hepatitis, Hodgkin’s lymphoma, granulomatous liver disease

## Abstract

Granulomatous hepatitis is a rare clinical entity characterized by granuloma formation in the liver, with a diverse etiology that includes infectious, autoimmune, and malignant causes. This case report details a 35-year-old male presenting with jaundice, abdominal pain, and malaise. Laboratory findings showed features of acute liver injury, with elevated liver enzymes and bilirubin, while imaging studies revealed hepatomegaly and lymphadenopathy. A liver biopsy confirmed non-caseating granulomas, with negative results for infectious and autoimmune etiologies. Further investigations, including a bone marrow biopsy, identified Hodgkin's lymphoma, establishing the diagnosis of granulomatous hepatitis as a paraneoplastic manifestation. The patient was referred for oncological treatment, underscoring the critical role of liver biopsy and histopathological evaluation in diagnosing granulomatous hepatitis of unclear origin. This case highlights the diagnostic complexities and the need for a multidisciplinary approach in identifying systemic malignancies presenting with hepatic manifestations.

## Introduction

Granulomatous hepatitis is a rare clinical entity characterized by granuloma formation within the liver parenchyma. While often associated with infectious or autoimmune etiologies, its occurrence as a manifestation of malignancy, particularly Hodgkin's lymphoma, is infrequent yet significant. Recent studies highlight that evaluating abnormal liver chemistries requires a multidisciplinary approach, integrating clinical, biochemical, and histopathological findings [1, 2]. Cases of granulomatous hepatitis linked to malignancies underscore the importance of liver biopsy in unexplained hepatic abnormalities, as it can reveal critical diagnostic insights [1, 3]. This report examines a case of granulomatous hepatitis, highlighting its diagnostic challenges and the importance of increased clinical vigilance in unusual presentations.

## Case presentation

A 35-year-old male, a merchant from Mexico City, presented to the emergency department with jaundice and abdominal pain. His medical history revealed a recent episode of fever, nausea, chills, and nocturnal diaphoresis, along with irritative urinary symptoms. He was initially treated for a urinary tract infection with ceftriaxone, nitrofurantoin, and symptomatic medications, but his symptoms persisted. On physical examination, he appeared jaundiced, with tenderness in the right hypochondrium and epigastrium. Laboratory tests showed significantly elevated liver enzymes (Aspartate Aminotransferase 179.8 u/l, Alanine Aminotransferase 197 u/l), bilirubin (total: 22.9 mg/dl, direct: 18.5 mg/dl), and alkaline phosphatase (621 u/l).

**Figure 1:**
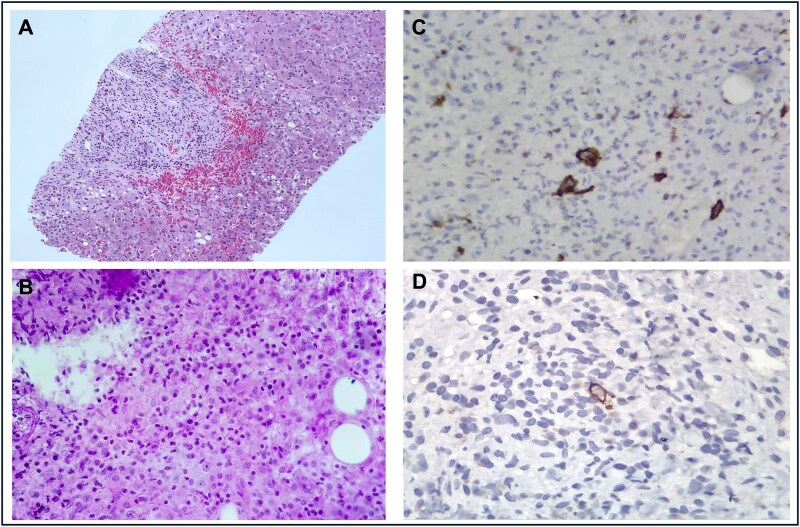
Histopathological analysis of liver tissue. (A) Hematoxylin and eosin (H&E) stain at low magnification shows granulomatous inflammation within the liver parenchyma. (B) H&E stain at high magnification reveals non-caseating granulomas of epithelioid macrophages and lymphocytes. (C) Special stains, including PAS and Ziehl-Neelsen, were negative for microorganisms, excluding infectious causes. (D) Immunohistochemical staining demonstrates positive markers consistent with Hodgkin's lymphoma, confirming the paraneoplastic origin of the granulomatous hepatitis.

Abdominal ultrasound revealed hepatomegaly, moderate hepatic steatosis, and gallstones without biliary duct dilation. A computed tomography scan of the abdomen demonstrated lymphadenopathy, hepatosplenomegaly, and no significant parenchymal abnormalities in the liver. Despite supportive care, his symptoms, including fever, continued. Given his worsening condition and the absence of an identifiable cause, a liver biopsy was performed. Magnetic resonance imaging was not pursued. The liver biopsy was image-guided and performed by the interventional radiology team, who selected the most accessible hepatic area based on ultrasound guidance, as there was no discrete lesion identified. The biopsy revealed non-caseating granulomas, and special stains ruled out common infectious agents, including tuberculosis and fungal infections (Fig. 1). A comprehensive workup to investigate the etiology of hepatic granulomas was initiated, including evaluation for systemic granulomatous diseases such as sarcoidosis and systemic lupus erythematosus, all of which were ruled out based on clinical, laboratory, and imaging findings. Viral serologies for hepatitis A, B, and C, as well as tests for autoimmune hepatitis, were negative. No other laboratory abnormalities—such as alterations in complete blood count, serum inflammatory markers, or lactate dehydrogenase—were present at that time to suggest an underlying malignancy.

Further diagnostic workup, including a bone marrow biopsy, identified hypercellular marrow infiltrated by classic Hodgkin's lymphoma cells, characterized by CD30+, CD15+, and LMP1+ markers. This confirmed the diagnosis of Hodgkin's lymphoma as the underlying cause of his granulomatous hepatitis. The granulomatous lesions were considered a paraneoplastic manifestation of the lymphoma.

Systemic corticosteroids were initiated to control hepatic inflammation; however, the patient’s condition progressively worsened. He subsequently developed disseminated intravascular coagulation and died shortly after the diagnosis of Hodgkin’s lymphoma was established.

## Discussion

Granulomatous hepatitis is a rare and challenging condition characterized by granuloma formation within the liver [1]. The diverse etiology encompasses infectious, autoimmune, drug-induced, and malignant causes. Hodgkin's lymphoma, while an uncommon trigger, can present as granulomatous hepatitis in a paraneoplastic context. Studies have reported granulomatous inflammation as the initial manifestation of Hodgkin's lymphoma, emphasizing the need for early histopathological evaluation in unexplained cases of granulomatous hepatitis [1, 4–6].

In some cases, granulomatous hepatitis coexists with hemophagocytic lymphohistiocytosis, adding further diagnostic complexity and underscoring the systemic nature of the inflammatory response [5]. Although the patient reported a prior episode of hepatitis, there was no evidence suggesting that it contributed to the current presentation of granulomatous hepatitis. The absence of chronic liver changes on histopathology, combined with negative viral and autoimmune markers, supports the conclusion that the previous hepatitis episode was unrelated to the current paraneoplastic condition. Non-Hodgkin's lymphoma can also mimic granulomatous hepatitis, with paraneoplastic cholestasis and hepatic granulomas presenting as extrahepatic manifestations [6]. These examples highlight the importance of immunohistochemistry and liver biopsy in elucidating the underlying pathology.

The differentiation between infectious and malignant causes often requires specialized diagnostic tools. Granulomatous hepatitis due to *Mycobacterium bovis* or *Mycobacterium avium* complex can mimic biliary diseases, necessitating the use of acid-fast bacillus staining or molecular diagnostic techniques for accurate identification [7]. Similarly, hepatic granulomatous inflammation caused by *Bartonella henselae*, particularly in immunocompromised individuals, emphasizes the importance of serology, Warthin-Starry staining, and PCR for diagnosis [8, 9]. Therefore, the diagnostic similarities between infectious diseases and granulomatous hepatitis related to malignancy highlight the necessity for a thorough and cooperative investigation approach.

As demonstrated in the present case, granulomatous hepatitis may be associated with paraneoplastic syndromes, such as those observed in non-Hodgkin's lymphoma [9]. In rare instances, these cases may require corticosteroid therapy to alleviate symptoms, as described in a patient with paraneoplastic cholestasis and granulomatous inflammation, where steroid treatment successfully resolved hepatic abnormalities [1, 5].

The prognosis of paraneoplastic granulomatous hepatitis remains uncertain and largely depends on the course of the underlying malignancy. While some case reports describe the improvement of hepatic abnormalities following corticosteroid therapy or effective cancer treatment, others have documented clinical deterioration despite such interventions [1, 5, 9]. Therefore, timely recognition and prompt initiation of targeted therapy are essential for optimizing outcomes in these patients.

In conclusion, granulomatous hepatitis requires a comprehensive diagnostic approach to identify its multifactorial etiology. This case underscores the importance of maintaining a high index of suspicion for systemic diseases, such as Hodgkin's lymphoma, when evaluating unexplained hepatic granulomas.
